# Dynamic Flow Approaches for Automated Radiochemical Analysis in Environmental, Nuclear and Medical Applications

**DOI:** 10.3390/molecules25061462

**Published:** 2020-03-24

**Authors:** Jixin Qiao

**Affiliations:** Department of Environmental Engineering, Technical University of Denmark, DTU Risø Campus, 4000 Roskilde, Denmark; jiqi@env.dtu.dk; Tel.: +45-4677-5367

**Keywords:** flow techniques, radionuclides, automation, radiochemical separation, environmental monitoring, nuclear emergency preparedness, radioactive waste characterization, medical isotope production

## Abstract

Automated sample processing techniques are desirable in radiochemical analysis for environmental radioactivity monitoring, nuclear emergency preparedness, nuclear waste characterization and management during operation and decommissioning of nuclear facilities, as well as medical isotope production, to achieve fast and cost-effective analysis. Dynamic flow based approaches including flow injection (FI), sequential injection (SI), multi-commuted flow injection (MCFI), multi-syringe flow injection (MSFI), multi-pumping flow system (MPFS), lab-on-valve (LOV) and lab-in-syringe (LIS) techniques have been developed and applied to meet the analytical criteria under different situations. Herein an overall review and discussion on these techniques and methodologies developed for radiochemical separation and measurement of various radionuclides is presented. Different designs of flow systems with combinations of radiochemical separation techniques, such as liquid–liquid extraction (LLE), liquid–liquid microextraction (LLME), solid phase extraction chromatography (SPEC), ion exchange chromatography (IEC), electrochemically modulated separations (EMS), capillary electrophoresis (CE), molecularly imprinted polymer (MIP) separation and online sensing and detection systems, are summarized and reviewed systematically.

## 1. Introduction 

Radiochemical analysis of natural and anthropogenic radionuclides plays an important role in (1) radioactivity monitoring in the environment and surroundings of nuclear installations; (2) nuclear emergency preparedness to identify the composition of a radioactive source and evaluate the impact of a nuclear accident/incident; (3) characterization of wastes from operations and decommissioning of nuclear facilities to ensure safe and cost-effective waste management; and (4) medical isotope production to achieve required purity and quality assurance. In all cases, rapid and effective radioanalytical approaches are desirable to cope with the growing demands of improving analytical speed and sample throughput and reducing labor intensity and cost. 

Flow analysis is considered as an efficient and universal chemical analysis method, which provides, usually, low sample consumption and possibilities of online sample processing in the flow system by effortless extension of the construction with additional units. Another essential feature of the flow analysis is its automation ability with full control over the fluid flow, volumes, flow rates, timing and detection conditions. This improves the analytical efficiency, provides satisfactory reproducibility and also minimizes human errors [1].

Many radiochemical analyses consist of a series of identical chemical separation steps with little or no variation from sample to sample, which makes them feasible for automation via the implementation of versatile flow techniques. A number of review papers have been published, focusing either on flow techniques for automation of certain radiochemical separation processes [2,3], development of radionuclide sensors [4] or methods for selected radionuclides/sample types [1,5,6,7]. This work presents the development and application of flow techniques for radiochemical analysis in different situations with focuses on technical design, assembly and performance of the flow systems. The application status, advantages, limitations and future perspectives for exploiting diverse flow systems in radiochemical analysis are critically reviewed. More than 100 publications were extracted mainly from data base websites such as the Web of Science, Google Scholar and Scopus from the 1950s until present, with keywords including “radionuclide,” “radiochemical analysis,” “automated,” “flow technique,” etc. Patents are not included for the review.

## 2. Basic Concept in Flow Analysis

**Figure 1:**
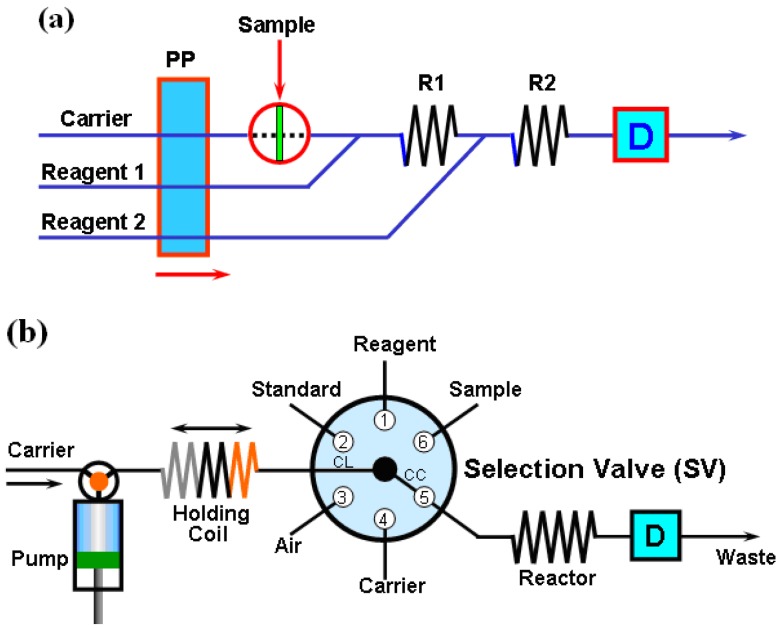
Diagram of a flow injection analysis (FIA) (**a**) and a sequential injection analysis (SIA) system (**b**) [19] (PP—peristaltic pump; R1, R2—reaction coils 1 and 2; D—detector).

The development of flow analysis laboratory methods began with the research of so-called segmented flow analysis (SFA) conducted in the 1950s by Skeggs [8], followed by significant technical progress in flow injection analysis (FIA) pioneered by Ruzicka and Hansen [9]. A basic FIA fluidic system is typically equipped with one peristaltic pump as the fluid driver, a tubing manifold, an injection valve with an injection loop to load a sample into the system and a detector. A schematic illustration of an FIA system is shown in Figure 1a. In more complex FIA setups there are also different modules for online sample processing incorporated into the flow systems. A sequential injection analysis (SIA) is considered to be a new generation of the FIA method, which, compared to FIA, can be considered more flexible because it introduces bidirectional flow and scales well for handling milliliter size to microliter size samples with precise control of volumes, flow rates and timing. An SIA system, as shown in Figure 1b, typically consists of a syringe pump, a multi-position rotary valve, a tubing manifold with a holding and a reactor coil and a detector. 

Over the past 50 years, flow techniques have been prompted in chemical analysis with the development of a number of highly specialized concepts, including multi-commuted flow injection analysis (MCFIA) [10,11], multi-syringe flow injection analysis (MSFIA) [12,13], multi-pumping flow system (MPFS) [14,15] and the recent lab-on-valve (LOV) and lab-in-syringe (LIS) systems [16,17,18]. 

## 3. Application of Flow Techniques in Radiochemical Analysis

The implementation of flow techniques for the determination of radionuclides is a relatively new and not very common field of application. The very first attempt at developing flow systems involving radiometric detection was proposed in the late 1960s for the determination of mercury in biological samples using neutron activation analysis (NAA) [20]. The term radiometric flow injection analysis (RFIA), relating to FIA systems combined with radiometric detectors, was suggested by Myintu et al. [21], and later extended to flow injection radiorelease analysis (FIRRA) and flow injection activation analysis (FIAA) [22]. The first study on RFIA constructed four types of radiometric cells using Geiger–Muller (GM) counters (end-window and liquid-type) and scintillation (NaI (Tl)) counters (cylindrical and well-type) for analysis of ^131^I and ^32^P [23]. The well-type scintillation (NaI(Tl)] cell was thereafter applied as a successful FIRRA for vanadate (V) determination by counting radioactive ^110m^Ag released through redox reaction between VO_3_^−^ and Ag (s) in a micro-column containing ^110m^Ag labelled silver [24]. 

Recent testing showed that flow techniques can be used for radiochemical analysis in many situations, as summarized in Table 1, including monitoring of environmental radioactivity for radiological risk assessment and remediation, nuclear emergency preparedness, characterization of radioactive materials in nuclear decommissioning and waste management, and production of radioactive isotopes for medical applications. 

Environmental radioactivity monitoring covers both the general environment and the surrounding environment of nuclear installations; e.g., nuclear power plants, nuclear waste storage facilities and disposal sites. In this case, sample types include environmental samples, such as air, precipitation, water, soil, sediment and biota, and effluents (e.g., waste discharges) from nuclear facilities. Environmental radioactivity monitoring focuses on both natural (e.g., ^210^Po, ^210^Pb, ^222^Rn, ^226^Ra and ^228^Ra) and artificial (e.g., ^3^H, ^14^C, ^89^Sr, ^90^Sr and actinides) radionuclides [25]. Typical analytical challenges involved in environmental radioactivity monitoring are trace or ultra-trace levels of radioactivity, large sample volume and large number of samples. 

For nuclear emergency preparedness, biological and environmental samples, including milk, urine, air, drinking water and soil are mostly analyzed. Radionuclides often required to be measured in emergency situations include ^89^Sr, ^90^Sr, ^137^Cs, ^239, 240, 241^Pu and ^241^Am [26,27]. The requirement of a rapid response and the unknown composition of radionuclides (interferences) are major challenges in such situations. 

For radioactive material characterization in nuclear decommissioning and waste management, constructional and operational materials (e.g., concrete, graphite, steel, ion exchange resin and coolant from nuclear reactors) are typically required to be analyzed for a number of radionuclides (e.g., ^3^H, ^14^C, ^36^Cl, ^41^Ca, ^55^Fe, ^63^Ni, ^90^Sr, ^99^Tc, Pu isotopes, ^241^Am and ^244^Cm) [28,29]. The large variations in radioactivity levels and sample matrix compositions occur often as challenges in the relevant radiochemical analyses. 

In medical isotope production, short-lived radioisotopes (e.g., ^18^F, ^64^Cu, ^99m^Tc, ^131^I, ^85^Sr, ^89^Zr, ^90^Y, ^68^Ga, ^188^Re, ^213^Bi) are produced in a cyclotron or nuclear reactor for diagnosis and treatment. Thorough radiochemical separation/analysis of the produced radioisotopes from the target materials (e.g., organic solvent or metal foil) is required to ensure their purity [30,31,32,33,34] and to monitor their entry into the environment [35,36]. 

## 4. Implementation of Flow Approaches in Radiochemical Analysis

**Figure 2:**
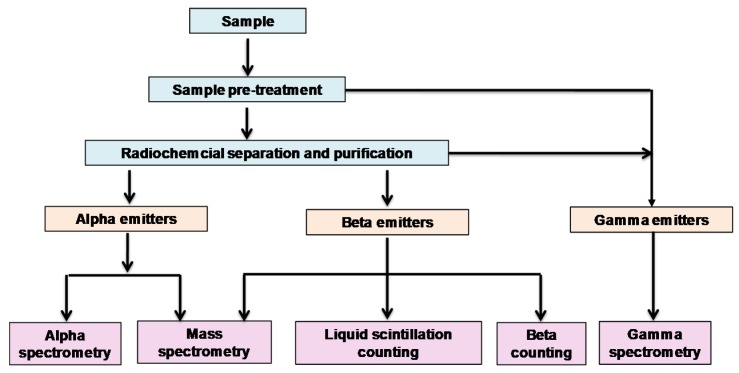
Schematic illustration of the overall procedure for radionuclide determination.

The overall procedure for radionuclide determination is presented schematically in Figure 2. Most gamma emitters can be directly measured by gamma spectrometers after suitable sample preparation (e.g., homogenization and packing). For alpha and beta emitters, so-called difficult-to-measure radionuclides, the analytical procedure can be divided into four steps: initial sample pretreatment, chemical separation/purification, source preparation and detection. Different approaches utilized in each step and their connections with flow approaches are discussed in the context with relevant examples taken from published articles. 

### 4.1. Sample Pretreatment 

Sample pretreatment is necessary to ensure sample homogeneity and appropriate conditions for quantification. Drying, grinding, sieving and ashing are often sequentially performed for solid samples when analyzing non-volatile radionuclides. For volatile radionuclides (e.g., ^3^H, ^14^C), fresh samples should be processed without drying and ashing, and for semi-volatile radionuclides (e.g., ^210^Po) ashing under high temperature should be avoided. For the extraction of most non-volatile radionuclides from solid samples—acid digestion using a mixture of mineral acids in open systems, with a pressure vessel or microwave assistance—is commonly applied [80]. Nevertheless, to ensure a complete release of radionuclides into the aqueous phase, alkaline fusion is often required to totally decompose the sample matrix. For liquid samples, preconcentration is performed either in-situ or in the laboratory. Typically, evaporation, co-precipitation or chelation can be used to remove most sample matrix elements. The evaporation involves reduction of sample volume by careful heating, whereas the selection of co-precipitation or chelation approach depends on the chemical property of the target radionuclide [81]. 

To study the dynamic release of ^226^Ra from phosphogypsum (PG), a lab-on-valve multi-syringe flow injection analysis (LOV-MSFIA) system was developed for the fully automated ^226^Ra lixiviation from PG [43]. The system coupled a homemade cell for online leaching of ^226^Ra, followed by preconcentration/purification of ^226^Ra using a renewable sorbent (MnO_2_) and its posterior co-precipitation with BaSO_4_. The BaSO_4_ co-precipitation was formed by dispensing Na_2_SO_4_ and acetate buffer/Ba^+2^ into the ^226^Ra fraction collector.

Online microwave assisted sample pretreatment incorporated in a sequential injection (SI) system was reported for ^99^Tc determination in nuclear waste [70]. The sample digestion was automatically performed using an open-vessel microwave digestion system. The flow reaction cell in the microwave system was constructed using concave-bottom digestion vessel. The automated fluid-handling system was configured using two syringe pumps equipped with the multi-position distribution valves. A two-way six-port injection valve was used to introduce the sample and two three-position selection valves were used upstream and downstream from the digestion cell to facilitate the delivery of sample/reagents and agitation gas to the reaction cell and uptake of digested sample to further sample purification on an anion exchange column. 

Despite numerous advantages offered by flow analysis, it is still rarely implemented in online sample pretreatment for radiochemical analysis. This might be related to the complicated sample pretreatment processes which are difficult to be fulfilled in a fully automated manner in flow systems. As a consequence, there is a lack of commercialized equipment with detailed procedures of such applications provided by manufacturers. This is also a bottle-neck in developing integrated and fully automated flow systems for practical implementation to process samples from their original phases. 

### 4.2. Chemical Separation and Purification 

Chemical separation and purification is often necessary for unambiguous and reliable quantification of individual radionuclides. In addition, concentrating analyte and removing matrix/interferences will typically improve sensitivity and detection limits. Individual, group or radionuclide/matrix separations represent an important part of the overall radionuclide determination scheme (Figure 2). Numerous operations in chemical separation and purification can be introduced into flow systems; e.g., liquid–liquid extraction (LLE), liquid–liquid microextraction (LLME), solid phase extraction chromatography (SPEC), ion exchange chromatography (IEC), electrochemically modulated separations (EMS), capillary electrophoresis (CE) and molecularly imprinted polymer (MIP) separation [37,38,41,42,48,52,58,64,69,79,82,83,84,85,86,87,88,89].

#### 4.2.1. Liquid–Liquid Extraction/Microextraction

Liquid–liquid extraction (LLE) is among the oldest of the preconcentration and matrix isolation techniques in analytical chemistry. LLE in a flow-based system can be carried out in a pipette tip, in-syringe, by a pseudo stationary phase or on a coating film consisting of the extractant adhered on an inert support. A flow-reversal wetting-film extraction approach towards the radionuclide separation in an SI system was reported for ^90^Sr determination in environmental samples [38]. The film coated on the walls of a tubular open reactor for selectively retained strontium ions was composed of 4,4´-(5´)-bis (tetra-butylcyclohexane)-18-crown-6 (BCHC) in 1-octanol. The noteworthy aspects of using a wetting-film phase instead of a solid-phase material are the reduction of crown ether consumption and the simplification of the operational sequence to avoid analyte carryover and reduce the resin capacity factor caused by irreversible interferences. A online LLE process for ^90^Y determination in environmental and biological samples has been carried out using a column containing di-2-ethylhexylphosphoric acid (HDEHP) adsorbed on a C18 support integrated in an MSFIA system [35]. In this way, the extraction process is carried out in a pseudostationary phase or a coating film which is generated by passing HDHEP solution through the column, and removed by washing the column with 96% ethanol.

**Figure 3:**
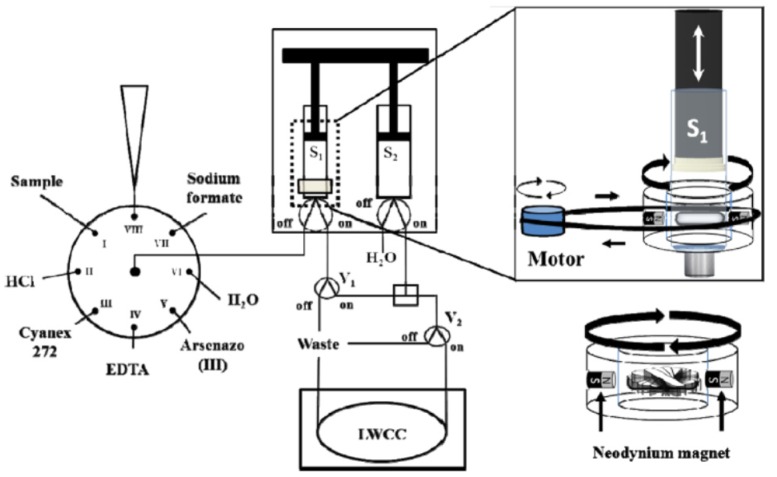
Schematic depiction of a flow system incorporating lab-in-syringe liquid–liquid microextraction (LIS-LLME) with magnetic stirring assistance (MSA) for radionuclide determination [47]. LWCC: liquid waveguide capillary cell, V: solenoid valve, S: syringe.

To improve the efficiency and cost-effectiveness of conventional LLE, liquid–liquid microextraction (LLME) and dispersive liquid–liquid microextraction (DLLME), among others, have been developed and applied in flow systems for radiochemical analysis. LLME is based on the usage of small volumes of organic solvents as extractants, which leads to high enrichment factors, even with limited sample volumes. A fully automated lab-in-syringe (LIS) LLME method (Figure 3) with magnetic stirring assistance (MSA) and spectrophotometric detection was developed and applied to U determination in environmental samples (soil, sediment, water and phosphogypsum) [47]. Uranium was extracted online and back-extracted with cyanex-272 in dodecane and hydrochloric acid, respectively, prior to reaction with arsenazo-III for the detection. A multisyringe burette coupled to a selection valve was used to implement the whole method, facilitating the U determination in a single instrumental assembly. The LIS technique permitted the simple automation of LLME methods with enhanced reproducibility and the capability of handling small volumes with satisfactory accuracy and precision.

DLLME is a fast microextraction technique based on the use of a ternary mixture, composed by an aqueous phase, an organic phase (extractant) and an additional organic solvent denoted as a disperser solvent, which is miscible in both phases. Extractant and disperser solvent are usually mixed and injected rapidly into the sample, producing a turbulent mixture due to the formation of small droplets of the extractant throughout the aqueous sample, thereby enhancing the effective surface area of extraction. This technique has attracted much attention due to its simplicity and the improved enrichment factors achieved. Furthermore, extraction times are usually short in DLLME since the extraction equilibrium is quickly reached due to the enhanced transfer area for the extraction. An approach exploiting LIS-DLLME for ^99^Tc extraction and preconcentration from biological samples (urine, saliva and liquid residues from treated patients) has been developed [36]. This system is very simple, comprising an eight-port multiposition selection valve connected to a multisyringe burette equipped with a 5 mL glass syringe. There are many other formats of LLE, yet they not often used in radiochemical analysis; e.g., direct-immersion single-drop microextraction (DI-SDME) and in-drop stirring SDME reported for the determination of nanomolar concentrations of lead using the automated LIS technique [90]. 

LLE and LLME offers the advantages of simplicity, flexibility and cost-effectiveness in flow systems for radiochemical analysis; however, they are deemed less selective and often require consecutive extraction, and thereby the analytical process is prolonged. Besides, hazardous organic liquid waste is generated during the analysis. Compared to the rapid development of chromatographic techniques, LLE and LLME are less popular in flow-based radiochemical analysis. 

#### 4.2.2. Chromatographic Separation 

##### Single-Column Chromatographic Separation

One of the first attempts to use flow injection based solid phase extraction chromatography (SPEC) for radiochemical separation was by Grate and co-workers [67]. The authors developed an SI system incorporating a Sr resin for determination of ^90^Sr in nuclear waste samples [67]. Later on they applied the SI system for actinides separated by TRU resin [74,76,77,91] and ^99^Tc by TEVA resin (Egorov et al., 1998). In the work for ^99^Tc determination [70,71], ion exchange chromatography (IEC) was also applied using macroporous anion exchange resin AGMP-1M through an implementation of a reversing elution, which ensured an effective separation process in a short time. 

**Figure 4:**
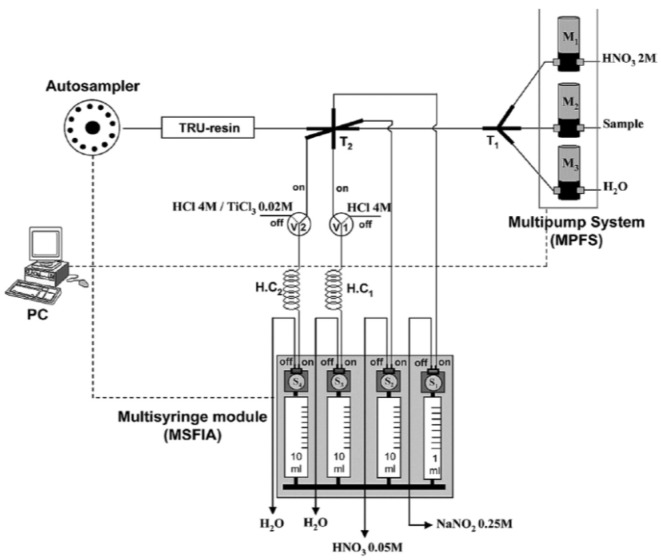
Schematic depiction of a multi-syringe flow injection analysis-multi-pump flow system (MSFIA– MPFS) [53].

For the determination of ^239^Pu, ^240^Pu and ^237^Np in environmental samples, SPEC using TEVA resin has been applied in SI systems to obtain high decontamination factors for interfering radionuclides, especially ^238^U [56,58]. Simultaneous determination of ^241^Am and ^239+240^Pu was reported by coupling SPEC using TRU resin in an MPFS (Figure 4), which is constituted of a multi-syringe buret equipped with four syringes as flow drivers [53]. Each syringe has a three-way solenoid valve at the head, which facilitates the application of multi-commutation schemes. The developed system was successfully used in analysis of real environmental and biological samples.

##### Tandem-Column Chromatographic Separation

**Figure 5:**
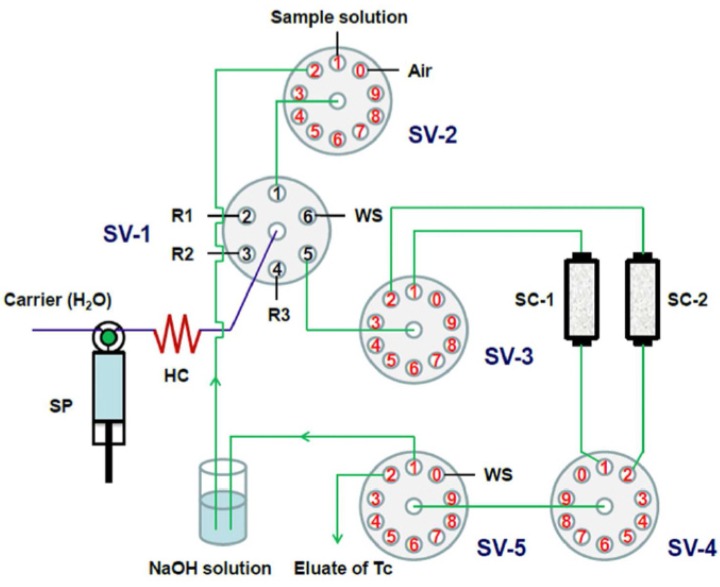
Schematic diagram of the sequential injection (SI) system coupling tandem chromatographic columns [41]. HC: holding coil, R: reagents, SC: separation column, SP: syringe pump, SV: selection valve, WS: waste.

In some cases, one chromatographic separation is not sufficient to purify the target radionuclides. Therefore, assembly of tandem-column chromatographic separation manifolds is necessary. For example, to improve the purification of ^99^Tc from large volume seawater samples, an SI method based on the use of two TEVA columns was developed [41]. The system consisted of one syringe pump as a flow driver and five selection valves for flexible connections between the two columns and for the delivery of samples/reagents (see Figure 5). Between the two column separations, a pH adjustment was performed via collecting Tc eluate (in 8 M HNO_3_) from the first TEVA column into a vial containing NaOH solution, in order to obtain a final solution of 0.1 M HNO_3_ for loading on the second TEVA column. An FI system for ^239^Pu and ^240^Pu determination in environmental samples was developed via tandem SPEC (Sr and TEVA resin) and online inductively coupled plasma mass spectrometry (ICP-MS) detection [49]. Sr resin in the first column was used to remove many interferences, including ^238^U, from the environmental sample, while the TEVA column was used to further remove ^238^U from Pu isotopes to eliminate its interference in the ICP-MS measurement. 

A tandem column purification method was also reported in medical isotope production, such as for the preparation of high-purity ^89^Zr (IV) oxalate [31] and purification of cyclotron-produced ^99m^TcO_4_^−^ [30]. In the ^89^Zr preparation system, the primary column was a microporous, strongly basic anion exchange resin onstyrene divinylbenzene co-polymer, while the secondary column was packed with hydroxamate resin. The ability to transfer ^89^Zr from one column to the next allows two sequential column clean-up to be performed prior to the final elution of ^89^Zr (IV) oxalate. In the ^99m^Tc purification system, triple tandem columns (SPEC packed with ABEC-2000, strong cation exchange (SCX) and aluminum (Al) columns) were applied to ensure a complete separation of ^99m^TcO_4_^−^ from MoO_4_^−^, wherein a mini-vacuum pump was used as the fluid driver [30].

**Figure 6:**
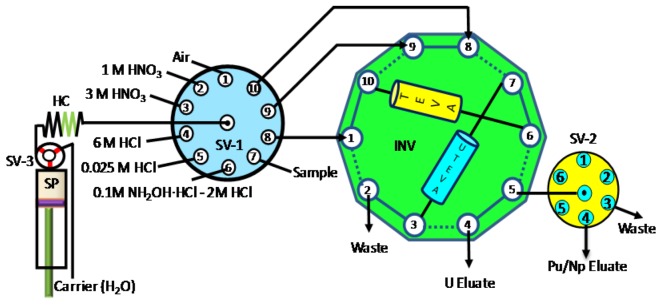
Schematic depiction of a sequential system (SI) incorporating a tandem-column for multi-radionuclide (Pu, Np and U) determination [63]. HC: holding coil, INV: injection vale, SV: selection valve, SP: syringe pump.

In many other cases, tandem-column systems provide advantages of sequential separation of multi-radionuclide from the same sample. For example, an SI system coupling a tandem TEVA and UTEVA column was reported for sequential separation of ^239,240^Pu/^237^Np and ^236^U in seawater [63]. After loading the sample onto the tandem TEVA/UTEVA column, the two columns were disconnected for further purification of Pu/Np on TEVA and U on UTEVA, respectively. The flexible connection of the two columns was realized via the use of a 10-port two-position injection valve (see Figure 6). An FI system was developed for the separation of ^238^Pu and ^90^Sr in seawater with the use of TEVA and Sr resin [54]. The sample was firstly loaded on the tandem TEVA/Sr resin; thereafter the Sr and TEVA column was manually switched in the system for further purification of ^238^Pu and ^90^Sr. 

In both single and tandem column chromatographic separations, essential problems related to the stability of the separation resins in multiple retention/elution cycles in flow systems were encountered in many works [35,57,58,67,69,77,78,87]. Even though the resin bead’s surface can be renewed chemically by washing with, e.g., complexing reagents or weak acids, the limited lifetime of each resin constrains its infinite reuse in the flow systems. Physical or chemical deformations of the resin during the regeneration process will deteriorate its separation performance (capacity, selectivity, etc.), leading to a carry-over effect, and influencing the flow dynamics in the flow system. For example, it was reported that TEVA resin could be reused up to 40 times for analyzing Pu isotopes in environmental soil (10 g), while after 20 times reuse, the flow system was forced to stop due to high backpressure caused by the compression of the TEVA column [58]. Therefore, in all cases of chromatographic separation, repacking columns with fresh separation material is necessary in order to ensure stable analytical performance of the flow systems.

##### Renewable-Column Chromatographic Separation

**Figure 7:**
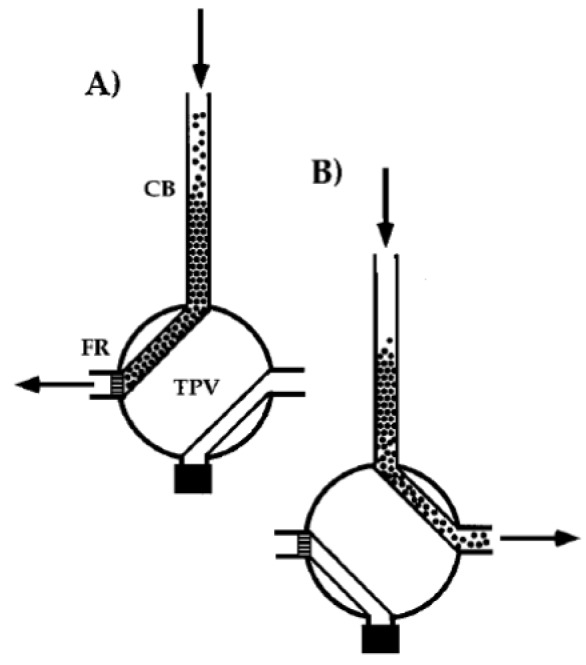
Schematic deposition of a renewable separation column (RSC) using a two valve [68] (**A**) column packing operation. (**B**) Disposal of separation material, CB: column body, FR: frit restriction, TPV: two-position valve.

Several renewable separation column (RSC) flow systems were developed with the aim of improving the analytical throughput. An RCS-SI system was reported for ^99^Tc water analysis, wherein the column was packed with a selective scintillating microsphere for absorbing and reacting with ^99^Tc for online detection [72]. The use of a dual-functional microsphere combined selective sportive and scintillating properties within a single bead. The microsphere in the column was renewed by fluidic replacement of the beads. A multipurpose SI system equipped with an RSC was developed for determination of different radionuclides in nuclear wastes [68]. Depending on the particular target analyte, the RSC was automatically packed with Sr resin for selective separation of ^90^Sr, TEVA resin for ^99^Tc or TRU-resin for ^241^Am. The RSC setup was controlled within a two-position valve, modified with a frit restriction, directly connected to the bottom of the column body (Figure 7).

**Figure 8:**
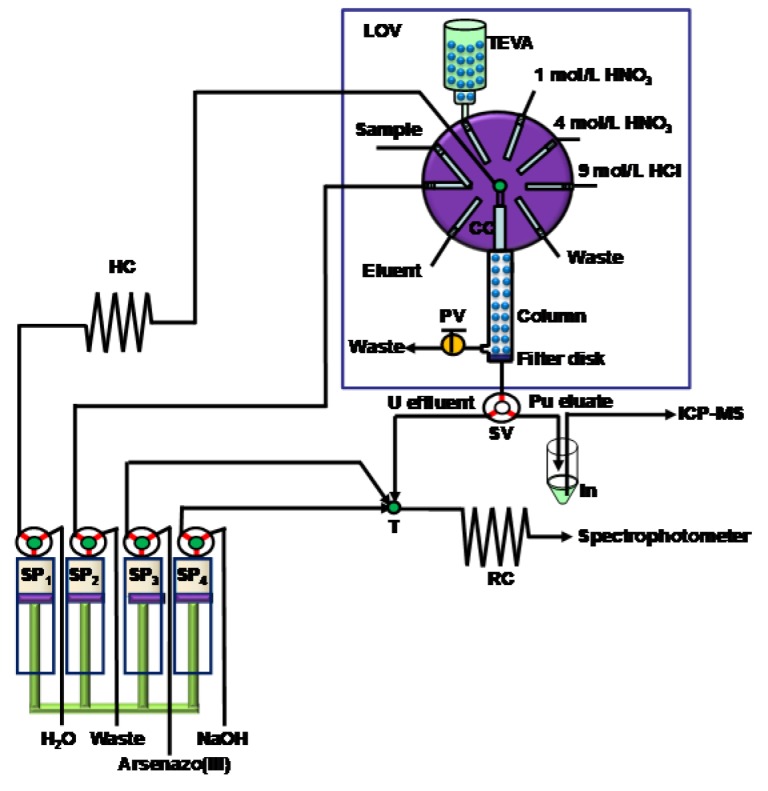
Schematic deposition of lab-on-valve (LOV) sequential injecting system renewable chromatographic separation [27]. HC: holding coil, PV: pinch valve, RC: reaction coil, SP: syringe pump, SV: solenoid valve, T: confluence point.

The LOV concept, introduced in 2000, allied to SIA, has emerged as an appealing downscaled analytical tool and provided more possibilities to renew the separation column in a flow system [17,18]. A number of LOV bead injection (BI) approaches have been applied for determination of actinides [27,45,48,65], ^99^Tc [42], ^226^Ra [44] and ^90^Sr [88]. The design of the LOV platform is normally based on a multi-port selection valve, where one upper port is connected to the reservoir of the separation material, and one lower port after certain modifications is used directly as the separation column or connected to an extended separation column (Figure 8). 

With the use of RSC, the time needed to change the resin and instrument conditions is saved. It provides the possibility for RSC flow systems to perform multi-sample or multi-radionuclide analysis in a consecutive manner with minimized carryover effect. The RSC format is typically miniaturized, favorable for cost-effective and efficient sample processing. 

##### Multi-Sample Chromatographic Separation

**Figure 9:**
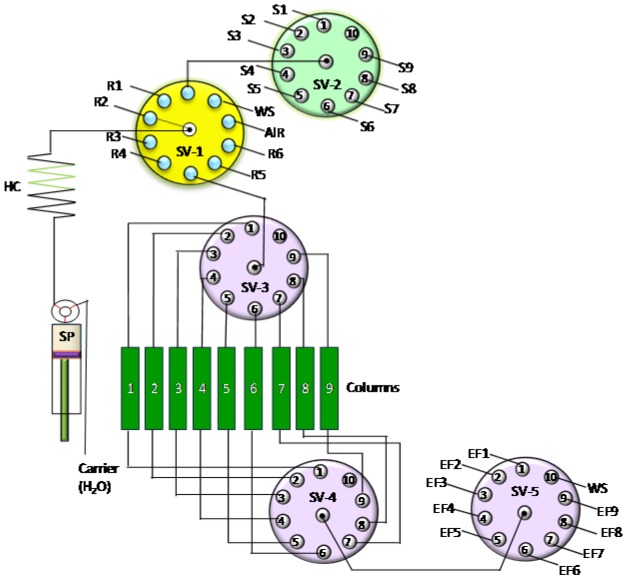
Schematic deposition of a flow system for four sample simultaneous processes [92]. SV: selection valve.

SI approaches coupling SPEC or IEC for processing nine samples in a sequential mode showed high sample throughput for ^239^Pu, ^240^Pu and ^237^Np environmental and biological assays [55,57,59,92]. The system (Figure 9) consists of one syringe pump as the fluid driver and five 10-port selection valves to integrate nine chromatographic columns (TEVA or AG 1 resin). A multi-sample processing FI system was developed for separation of ^239+240^Pu, ^210^Po and ^210^Pb in environmental samples [50]. The separation was conducted in two parallel lines for two samples, which was respectively applied to Pu with Dowex 1× 8 anion exchange resin, and ^210^Po and ^210^Pb with Sr resin in an independent sequence.

**Figure 10:**
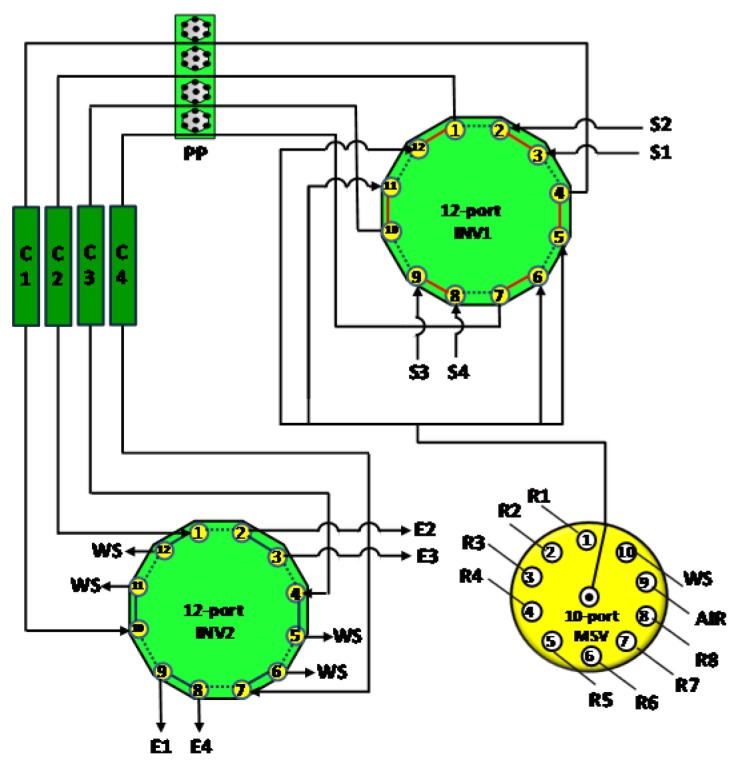
Schematic deposition of a flow system for four-sample simultaneous processing [64,79]. SV: selection valve.

A modular automated radionuclide separator (MARS) has been manufactured and applied to determine ^99^Tc in groundwater and ^89^Sr/^90^Sr in milk samples [26,62]. The separator is capable of processing four samples in parallel with four integrated SPEC columns (TEVA or Sr resin). The separator consists of a four-channel peristaltic pump as the fluid driver, a 6-port selective valve for selecting different reagents, a 5-way flow distribution connector to distribute reagents into the four separation lines, four 2-port selection valves to select sample or reagent delivered to the columns and four 3-way distribution valves to select eluate/waste after the column separation. A multi-sample processing flow system simultaneously handling four samples has also been used for the determination of ^239^Pu, ^240^Pu, ^237^Np, ^236^U, ^238^U and ^99^Tc in seawater [64,79] (Figure 10). The system is more compact via the use of two 12-port injection valves, in the front and bottom end of the chromatographic columns to facilitate the respective selection of sample/reagent and eluate/waste. A semi-automated method handling eight samples in parallel has been reported for monitoring ^89^Sr, ^90^Sr and ^226^Ra in milk and drinking water samples [39]. The method used a 2-stage purification process during which the first purification step using strong cation exchange (SCX) chromatography was performed within an FI system, followed by the second purification using high-performance ion chromatography (HPIC). 

The flow-based multi-sample processing methods alleviate the analytical workloads compared to error-prone and batch-wise manual methods. The application of automation is important for obtaining good analytical repeatability and constant sample throughput. One drawback related to the use of a peristaltic pump in the FI systems could be the aging/deformation of peristaltic pump tubing, resulting in changes of flow rate during operation. Therefore, precalibration of flow rate is necessary each time prior to the analysis, which could be avoided by replacing peristaltic pumps with multichannel syringe pumps in the flow systems. 

#### 4.2.3. Other Separation Methods

An FI system employing online electrochemically modulated separations (EMS) was developed for determination of Pu isotope ratios [66]. The flow-through voltammetric cell was used to accumulate Pu by anodic oxidation of Pu(III) to Pu(IV and VI), and then to release them at a controlled potential. Due to more negative potentials being required for U(IV), the separation of Pu from the U interference was possible. Capillary electrophoresis (CE) in combination with ICP-MS has been used for the separation of Pu ions in the oxidation states III-VI and Np ions in the oxidation states IV and V. The method was applied to study the redox behavior of Pu in a natural groundwater rich in humic substances under anaerobic conditions, providing advantages of short separation time and a high separating efficiency [75].

Molecularly imprinted polymers (MIPs) are widely regarded as ideal recognition elements for sensor applications because of their stability, selectivity and affinity [93]. Metal ion imprinting (IIP), based on molecular imprinting technology, is used for preparing materials that can recognize metal ions. Proof-of-concept applications of IIP materials for radionuclide separation have been reported; e.g., the selective removal of ^60^Co from wastewater [94] and selective extraction of ^90^Y and ^152^Eu for medical applications and nuclear power plant monitoring [95]. Yet, MIP as a solid phase extraction (SPE) reactor in a flow setup has not been applied to the radioanalytical field [96,97].

### 4.3. Detection of Radionuclides

The detection of radionuclides is normally based on quantifying their characteristic radiations, i.e., radiometric methods, or directly courting their atoms, i.e., mass spectrometric methods. In some cases, radionuclides can also be determined spectrophotometrically based on their reactions with complexing agents. 

#### 4.3.1. Radiometric Detection

Radiometric detection techniques which have been applied in flow systems include proportional counter, ionization chamber and liquid scintillation counter (LSC) for alpha emitters; Geiger–Müller counters; LSC and Cerenkov cells for beta emitters; and gamma spectrometry for gamma emitters [1]. The first online detection for ^90^Sr was based on the use of flow-through LSC in an SI system, wherein the purified ^90^Sr after chromatographic separation was mixed with scintillation liquid and transported to the LSC [67]. Stopped-flow mode LSC detection was reported for online measuring of ^99^Tc in an SI system [69].

A sensor device integrating LSC has been developed for analysis of ^99^Tc in groundwater [72]. Dual function sensor beads or the mixture of sorbent (TEVA resin) and scintillator beads were arranged in a mini-column located between the two photo-multiplier (PM) tubes of the scintillation detection system. Upon retention of pertechnetate ions on the resin, the scintillation pulses produced by the radioactive decay of ^99^Tc are counted. The detection absolute efficiency was 56%, which is sufficiently high for a practical analytical application. This composite bed approach also allows the use of SPE sorbents that can not be readily converted to scintillators by impregnation techniques. A mini-column sensor with the use of a packed bed containing a mixture of anion-exchange resin and scintillating plastic beads was also applied for ^99^Tc online detection in water [40]. 

**Figure 11:**
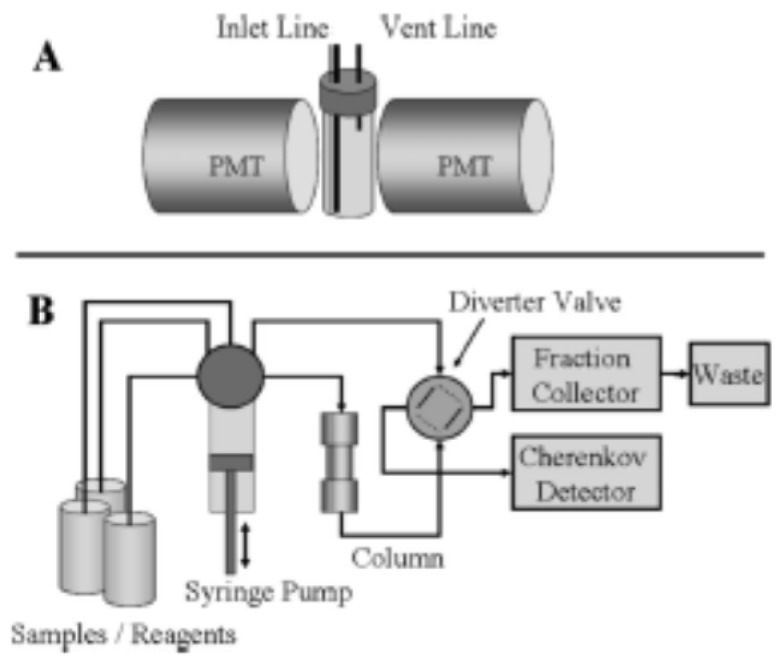
Schematic deposition of a flow-system-coupling Cherenkov detection flow cell configured between dual photo-multiplier tubes (PMTs) [37].

An automated fluid handling system coupled to a Cherenkov radiation detector for measuring ^90^Sr via the high-energy decay of its daughter, ^90^Y, has been assembled and applied to Hanford groundwater analysis [37]. A SuperLig 620 column in the system enables preconcentration and separation of ^90^Sr in the sample, and creates a pure ^90^Sr source from which subsequent ^90^Y ingrowth can be measured. ^90^Y is fluidically transferred from the column to the Cherenkov detection flow cell configured between dual PM tubes for quantification and calculation of the original ^90^Sr concentration (Figure 11). 

A prototype apparatus for at-line/online monitoring of ^99^Tc in nuclear wastes demonstrated an analytical turnover time of less than 15 min. [71]. The apparatus integrates microwave-assisted sample preparation, anion exchange column separation and detection with a flow scintillation detector in one fully automated sequence. The authors used standard addition method to ensure a matrix-matched measurement to calibrate the process. The ^99^Tc standard was delivered by a syringe pump to the digestion vessel immediately following sample delivery to the microwave digestion chamber. Standard addition was automatically performed after every sample during research and after every fourth sample during extended monitor operation. 

Even though radiometric detection techniques are being willingly introduced to flow analysis, they are mostly applied to online monitoring of high radioactive samples for nuclear waste management. For low-level environmental samples, the radiometric measurements are used mostly offline due to the requirement of relatively long counting time. 

#### 4.3.2. Mass Spectrometry

With the rapid development of mass spectrometry, especially ICP-MS, its application for the online detection of radionuclides is being prompted. The unique advantages of using ICP-MS include short analytical time (within several minutes), multi-radionuclides measurement capability and simple source preparation which facilitates complete automation of the determination processes in versatile flow systems. 

A number of examples in the literature demonstrate ICP-MS as a widespread detection technique applied in flow analysis [51,52,60,76,78,98,99]. The introduction of FI-ICP-MS in 1986 opened the way to automate classical ICP-MS methods using FI techniques [100]. The first effective attempt to design a flow system coupling ICP-MS for radionuclide analysis was intended for the determination of ^99^Tc, ^230^Th and ^234^Th in soil [73]. The radionuclides were separated from matrix components with TEVA and TRU resins in the FI system and then transported directly to an ICP-MS detector. 

The use of a highly specialized ICP-MS instrument enhanced the sensitivity significantly, especially for long-lived radionuclides, such as ^99^Tc and actinides, allowing for precise trace and ultra-trace level radiochemical analyses. An FI system with online preconcentration/separation with TEVA resin and ICP-MS detection enabled the completion of a Pu urine assay in 15 min. with a detection limit of 0.2 mBq/L for ^239^Pu and ^240^Pu [85]. An FI-ICP-MS system developed for ^99^Tc determination in soil achieved a detection limit of 50 mBq/L ^99^Tc and an analytical turnover time of 3–5 h [78]. 

Despite the numerous advantages of ICP-MS mentioned above, some limitations of this method should be noticed, such as sensitivity to spectral and non-spectral interference; e.g., ^238^U to ^238^Pu, ^238^U^1^H to ^239^Pu, ^99^Ru and ^98^Mo^1^H to ^99^Tc. Currently, the majority application of FI-ICP-MS is still limited to long-lived radionuclides; e.g., ^99^Tc and actinides. For detection of short-lived radionuclides, e.g., ^90^Sr, a higher detection limit inherent to ICP-MS is a disadvantage compared to radiometric methods. The method detection limit (MDL) for ^90^Sr has been reported to be 14.5 Bq/L (which was sufficient for ^90^Sr determination in real nuclear reactor coolant) with online ICP-MS detection connected in a LOV-SI system [61].

#### 4.3.3. Spectrophotometric Measurement

The basis of spectrophotometric measurements is usually the reaction of a determined element with a complexing agent. This leads to formation of a color product, detectable by a conventional spectrophotometer. The most reported flow systems coupling spectrophotometric detection were applied to determination of U and Th. For example, an FI method with online spectrophotometric detection was successfully applied to U determination in seawater [46]. Prior to spectrophotometric detection, the seawater sample injected into the FI system was concentrated with a column packed with styrene-divinylbenzene copolymer resin (Bio-Beads SM-2) modified with dodecylamidoxime. The system provided very high sample throughput (23 per hour) and chemical yield (95% to 99%).

A hyphenated LOV-MSFIA system, coupled to a long path length liquid waveguide capillary cell (LWCC), performed spectrophotometric determination of U and Th in different types of environmental samples [45]. Online separation of U and Th is carried out in a UTEVA column, which is automatically regenerated within the LOV platform. Following the separation, U and Th are spectrophotometrically detected after reaction with arsenazo-III. 

### 4.4. Automation of Flow Systems

During the development of a prototype device or a virtual instrument, the indispensable requirement for the automation of any flow systems relies on expertise in mechanical design and assembly, electronics and software development. The automation of most flow systems presented in this work generally is fulfilled via two approaches: (1) assembly of flow systems based on commercially available integrated flow setups or individual components (e.g., valves and pumps), including electronic control modules, in combination with commercial software for instrument control and data acquisition. (2) The design and assembly of flow systems based on commercial components, in combination with in-house developed electronic boards, instrument controls and data acquisition software. 

At present there are several commercialized software for flow systems, including Perkin Elmer AA Winlab [89,101,102], Atlantis [67], FIAlab [56,58,68,69,72,77,87,90], etc. One of the main constraints in most commercial software packages is the high specificity for a certain configuration. To avoid the inconvenience, some in-house developed software based on LabVIEW [26,62,82], LabWindows [37,70,103,104] and Delphi plus Visual C++ (Autoanalysis software) [12,27,36,42,43,44,45,47,53,61,105,106] respectively, has been widely applied in the radioanalytical field. In most cases of online radionuclide detection, signals from the detectors (e.g., LSC and ICP-MS) are directly collected and processed by standalone software associated with the detection instruments. It is still not common that radionuclide signals are directly digitized by the computer-based digital oscilloscope card and processed by the same software for controlling the flow system. Besides, most software is prioritized toward mechanical control of the flow systems, whereas an integrated software package controlling the whole radioanalytical procedure covering sample pretreatment, chemical separation, detection and data processing is limited.

### 4.5. Perspectives on the Future Development of Flow Approaches for Radiochemical Analysis

Table 1 presents an overview of the applications of flow approaches in radiochemical analyses in different fields as well as their analytical performances in terms of chemical yield, limit of detection, repeatability, precision and turnover time. Even though it is not very straightforward to set benchmarks for different flow techniques, as they are applied in different situations with different analytical criteria, most flow approaches demonstrate satisfactory sensitivity, cost-effectiveness, robustness and efficiency under the circumstances presented in the original research. For medical applications, most applications of flow approaches serve as platforms for automated separation and purification of the produced radioisotopes, rather than radiochemical analysis.

It is noted that most applications are focused on “classical” radionuclides with the majority consisting of actinides and fission products (e.g., ^99^Tc, ^90^Sr) appearing in environmental monitoring, emergency preparedness and nuclear industry. In recent years, more and more countries launched comprehensive plans for decommissioning of nuclear installations, including nuclear power plants, research reactors and nuclear fuel reprocessing facilities [107,108,109,110,111,112]. It is apparent that the heavy demands in nuclear decommissioning and waste management require solid technical support for sample characterization to facilitate the categorization and management of radioactive waste. Demands in determining hard-to-measure radionuclides, especially for several newly-appearing long-lived radionuclides (e.g., ^93^Zr, ^93^Mo, ^79^Se, ^126^Sn, ^135^Cs) in nuclear decommissioning are becoming more and more notable [113]. To achieve economical and efficient waste characterization, the development of effective flow based radioanalytical methods for wider ranges of radionuclides and sample types (e.g., construction materials of nuclear reactors) is necessary.

In many applications, it is desirable to perform measurements on-site or in-situ using analyzer or sensor instruments. This sets a high requirement for radionuclide analyzers: to perform all the functions carried out in the laboratory rapidly and efficiently in an automated device. Current flow systems developed for radiochemical analysis are primarily laboratory-based, which are not well suited to real-time monitoring. Challenges still exist for creating a promising prospect for applying flow techniques to on-site or in-situ monitoring of radioactive content, such as (1) having an automated sample pretreatment process; (2) creating a highly selective purification approach; and (3) ensuring quick online detection with sufficiently low detection limits via portable detectors. Besides, the compactness, robustness and flexibility of the system are also important to ensure the mobility and applicability in real circumstances. To tackle current limitations in the automation of radiochemical analysis, development of thoughtful flow approaches in combination with state-of-the-art technologies, such as microfluidics, artificial intelligence, big data and neural networks should be considered.

## 5. Conclusions 

Flow analysis is a useful tool in the hands of analysts and it improves the determination of radionuclides by constructing various configurations of flow systems with satisfactory effectiveness. Versatile flow approaches have been utilized in different steps for radiochemical analysis, including sample pretreatment, chemical separation/purification, source preparation and detection. The automation of the analysis leads to improvement of the functional parameters by increasing the reliability of the results and reducing the duration of measurements, and makes the analysis less laborious and safer because of less exposure to radioactivity.

Nevertheless, continuous development of more advanced flow approaches is necessary to cope with the growing demands for radiochemical analysis in different fields, especially nuclear decommissioning, considering not only “classical” but also “emerging” radionuclides and sample types. It is also desirable to develop smart and cost-effective real-time monitoring mobile devices for online chemical processes and detection, with the possibility to transmit results using wireless communication to a central server where the data could be stored and analyzed. 

## Figures and Tables

**Table 1:** Overview of flow approaches developed for radionuclide determination.

Purpose	Radionuclides	Sample Type	Flow System Design	Sample Processing Mode	Chemical Separation	Measurement Technique	Performance	Ref
Environmental radioactivity monitoring	^90^Sr	Groundwater (0.35 L)	FI	Single sample	SuperLig 620 column	LSC (Cherenkov counting)	Chemical yields: 99.9 ± 2.8% LOD: 0.057 Bq/L Turnover time: 41.5 h (27 h for ^90^Y ingrowth and 13.5 h for counting)	[37]
	^ 90 ^ Sr	Water, powdered milk, soil (2 mL of sample solution)	SI flow-reversal wetting-film extraction	Single sample	Wetting-film of BCHC in 1-octanol	LBPC *	Applied to measure ^90^Sr ranging in 0.07-0.30 Bq Chemical yield: up to 80% Precision: <3% RSD (*n* = 10)	[38]
	^89^Sr, ^90^Sr, ^226^Ra	Milk (1000 mL), water (800 mL)	Semi-automated FI combined with HPLC	Multi-sample (8 samples)	Cation exchange chromatography (16 mL of Dowex 50W-X8) + HPIC (PRP-X400 poly (styrene–divinylbenzene)-sulfonate cation-exchange)	LSC *	Chemical yield: >95% for Sr, ca.100% for Ra MDC: 30 mBq/L of ^89^Sr, 20 mBq/L of ^90^Sr, 2 mBq/L of ^226^Ra Turnover time: 4–5 h	[39]
	^ 99 ^ Tc	Groundwater (150 mL)	SI-minicolumn sensor	Single sample	Anion exchange chromatography (AG 4 × 4)	Flow-through scintillation counter	-	[40]
	^ 99 ^ Tc	Seawater (50–200 L)	SI	Single sample	Tandem extraction chromatography (two 1.5-mL TEVA columns)	ICP-MS *	Chemical yield: 60–75% LOD (200 L seawater): 7.5 µBq/L of ^99^Tc Turnover time: 24 h (for a batch sample (n > 4))	[41]
	^ 99 ^ Tc	Soil (0.5 g), water (0.1–100 mL)	LOV-SI	Single sample Renewable column	Extraction chromatography (32 mg TEVA resin)	ICP-MS *	Chemical yield: 94–98% LOD: 5 pg of ^99^Tc Precision: 3.8% (*n* = 5) Repeatability: 2% (*n* = 10) Turnover time: 2–5 h	[42]
	^226^Ra	Leachate from phosphogypsum	LOV-MSFIA	Single sample Renewable column	MnO_2_ coated on macroporous bead cellulose (0.3 g)	LBPC *	Chemical yield of ^226^Ra: > 90%	[43]
	^ 226 ^ Ra	Drinking, natural water	LOV-MSFIA	Single sample Renewable column	MnO_2_ coated on macroporous bead cellulose	LSC * LBPC *	Chemical yield: > 90% MDA: 4 mBq/L (LSC), 20 mBq /L (LBPC) Precision: 1.7% RSD Turnover time: 20 min	[44]
	^232^Th, ^238^U	Sediment, water (sample solution up to 30 mL for U, up to 8 mL for Th)	LOV-MSFIA	Single sample Renewable column	Extraction chromatography (0.03 g UTEVA)	Spectrophotometry with arsenazo-III	LOD: 5.9 ng/L of U, 60 ng/L of Th. Repeatability: 1.6% (*n* = 10) Turnover time: 11–50 min for U, 10–20 min for Th	[45]
	^238^U	Seawater (10 mL)	FI	Single sample	Styrene-divinylbenzene copolymer resin, Bio-Beads SM-2	Spectrophotometry with Chlorophosphonazo III	Chemical yields: 95–99% LOD: 130 ng/L Turnover time: 2.6 min	[46]
	^ 238 ^ U	Soil, sediment, water, phosphogypsum	LIS-MSA-MSFIA	Single sample	LIS-LLME	LWCC spectrophotometry	Chemical yield: close to 100% LOD: 3.2 mg/L. Precision: 3.3% RSD	[47]
	^238^U	Phosphogypsum, sediment, water	LOV-MSFIA	Single sample Renewable column	Extraction chromatography (0.03 g UTEVA)	Spectrophotometry with arsenazo-III	Chemical yield: > 90% LOD: 10.3 ng/L of U. Repeatability: 1.6% (*n* = 10) Turnover time: 11–50 min	[48]
	^ 239 ^ Pu, ^240^Pu	Soil and sediment (0.5–1 g)	FI	Single sample	Tandem chromatography (0.5 mL Sr resin and 0.17 mL TEVA resin)	ICP-MS	Chemical yield: > 70% LOD: 9.2 mBq of ^239^Pu, 25 mBq of ^240^Pu and 0.87 mBq of ^242^Pu Turnover time: 5 h	[49]
	^239+240^Pu, ^210^Po, ^210^Pb	Soil (10 g), phosphogypsum (0.5 g)	FI	Multi-sample (2 samples)	Anion exchange and extraction chromatography (Dowex 1 × 8 resin, 100–200 mesh and Sr resin)	Alpha spectrometry * LSC *	Chemical yield: 87 ± 8% for Pu, 86 ± 6% for ^210^Pb, 82 ± 6% for ^210^Po Turnover time (online separation): 4.8 h for ^210^Po and ^210^Pb, 5.0 h for Pu	[50]
	^ 239 ^ Pu, ^240^Pu	Seawater (1 L)	FI	Single sample	Co-precipitation and ion exchange *	ICP-MS	LOD: 5 mBq/L Precision: 12% RSD	[51]
	^ 239 ^ Pu, ^240^Pu	Seawater (3–10 L)	FI	Single sample	Tandem chromatography (Sr resin and TEVA resin)	ICP-MS	LOD: 1.5 mBq/L of ^239^Pu, 1.6 mBq/L of ^240^Pu Precision: <3.4% RSD (*n* = 7) for ^239^Pu and < 5% RSD (*n* = 7) for ^240^Pu Turnover time: 4 h	[52]
	^239+240^Pu, ^241^Am	Soil, vegetable ashes leachate, urine, blood	MSFIA-MPFS	Single sample	Extraction chromatography (0.08 g TRU)	Low- background proportional counter	Chemical yield: <90% for both Pu and Am LOD: 4 Bq/L Precision: 3% Turnover time (online separation): 40 min.	[53]
Environmental radioactivity monitoring, nuclear emergency preparedness	^ 90 ^ Sr, ^238^Pu	Seawater (1 or 10 L)	FI	Single sample	Tandem chromatography (4 or 35 mL Sr resin and 4 or 6 mL TEVA resin)	LSC * Alpha spectrometry *	Chemical yield: 87.8 ± 6.5% for Sr, 62.5 ± 10.4% for Pu Turnover time (online separation): 3.2 h for 1 L seawater, 9.4 h for 10 L seawater	[54]
	^ 237 ^ Np	Soil/sediment (1-10 g) and seaweed (20 g)	SI	Nice samples in sequential mode	Anion exchange chromatography (2 mL AG 1 × 4 resin)	ICP-MS *	Chemical yield: 60–70% for Np Turnover time (in-line anion exchange chromatography): <2.5 h	[55]
	^ 237 ^ Np, ^239^Pu ^240^Pu	Soil (10 g) and seaweed (20 g)	SI	Single sample	Extraction chromatography (2 mL TEVA resin)	ICP-MS *	Chemical yield: 80–105% LOD (for 10 g soil): 1.5 mBq/kg of ^239^Pu, 5.3 mBq/kg of ^240^Pu, 16 mBq/kg of ^237^Np Turnover time (in-line extraction chromatography): <1.5 h	[56]
	^ 237 ^ Np, ^239^Pu, ^240^Pu	Soil/sediment (0.5–100 g) and seaweed (20 g)	SI	Nice samples in sequential mode	Anion exchange chromatography (2 mL AG MP-1M resin)	ICP-MS *	Chemical yield (100 g soil): 85 ± 10% for Pu, 79 ± 10% for Np Turnover time (in-line anion exchange chromatography): <3.5 h	[57]
	^ 239 ^ Pu, ^240^Pu	Soil/sediment (10–200 g), seaweed (20 g), seawater (200 L)	SI	Single sample	Extraction chromatography (2 mL TEVA resin)	ICP-MS *	Chemical yield: 80–105%DFs for U, Th, Hg and Pb: > 10^4^. Duration for in-line extraction chromatography: <1.5 h	[58]
	^ 239 ^ Pu, ^240^Pu	Soil/sediment (5–100 g), seaweed (20 g)	SI	Nice samples in sequential mode	Anion exchange chromatography (2 mL AG 1 × 4 resin)	ICP-MS *	Chemical yield: up to 90% Turnover time (in-line anion exchange chromatography): <2.5 h	[59]
Environmental radioactivity monitoring, nuclear safeguards	^ 238 ^ U, ^242^Pu	Urine (1 mL) and tap water (10 L)	FI	Single sample	Co-precipitation and extraction chromatography (TEVA) for water sample *	ICP-MS	LOD: 0.09 fg of ^238^U and 0.015 fg of ^242^Pu	[60]
Environmental radioactivity monitoring, nuclear waste management	^ 90 ^ Sr	Rain water and reactor coolant	LOV-MSFIA	Single sample Renewable column	Extraction chromatography (0.35 mL Sr resin)	ICP-MS	Chemical yield: 53–100% Turnover time: 16–24 min for 5 mL sample, 60 min for 100 mL sample, 6 h for 1 L sample	[61]
	^ 99 ^ Tc	Ground water (250 mL)	FI	Multi-sample (4 samples)	Extraction chromatography (1.4 g TEVA resin)	ICP-MS *	Chemical yield: 96 ± 2% LOD: 0.2 ng/L ^99^Tc Turnover time: 81min	[62]
Environmental radioactivity monitoring, nuclear safeguards, radioecology and tracer studies	^ 236 ^ U, ^237^Np, ^239^Pu, ^240^Pu	Seawater (10 L)	SI	Single sample	Tandem chromatography (2 mL TEVA resin and 1 UTEVA resin)	ICP-MS * AMS *	Chemical yields: 70–100% Turnover time: 8 h	[63]
Environmental radioactivity monitoring, emergency preparedness, radioecology and tracer studies	^ 99 ^ Tc, ^237^Np, ^239^Pu, ^240^Pu, ^238^U	Seawater (200 L)	FI	Multi-sample (4 samples)	Extraction and anion exchange chromatography (TEVA, AG MP-1M, UTEVA resin)	ICP-MS * AMS *	Chemical yield: 50–70% LOD: 8 µBq/L of ^99^Tc, 0.26 nBq/L of ^237^Np, 23 nBq/L of ^239^Pu, 84 nBq/L of ^240^Pu and 0.6 µBq/L of ^238^U Turnover time: 3–4 day	[64]
Medical isotope production	^ 89 ^ Zr **	Cyclotron bombarded Y foil	SI	Single sample	Tandem chromatography (AG MP-1 M and hydroxamate resin)	Gamma spectrometry	Chemical yield: 95.1 ± 1.3%	[31]
	^ 90 ^ Y	Water, urine and blood	MSFIA coupling online column-based LLE	Single sample	LLME in a column (0.32 mL) containing HDEHP absorbed on C18 (0.11 g)	LBPC *	Chemical yield: 100 ± 2.3% (*n* = 10). LLD: 5 mBq of ^90^Y	[35]
	^ 99 ^ Tc	Urine, saliva and hospital residues	LIS-DLLME	Single sample	LIS-DLLME with 22.5% of Aliquat^®^336 in acetone	LSC *	MDA: 75 mBq Turnover time (extraction): 7.5 min	[36]
	^ 99m ^ Tc	Cyclotron bombarded Mo target	Vacuum pumping flow system	Single sample	Triple tandem chromatography (ABEC-2000, SCX and Al resin)	Gamma spectrometry	Chemical yield: close to 90% Turnover time: 27 ± 2 min	[30]
	^ 68 ^ Ga, ^99m^Tc, ^188^Re, ^213^Bi **	Parent radionuclides ^68^Ge for ^68^Ga, ^99^Mo for ^99m^Tc, ^188^W for ^188^Re, ^225^Ac for ^213^Bi	SI	Single sample	Tandem chromatography ^68^Ge/^68^Ga: 50W × 8 +UTEVA ^99^Mo/^99m^Tc: ABEC − 2000 + 50W × 8/Diphonix^188^W/^188^Re: ABEC − 2000 + 50W × 8/Diphonix^225^Ac/^213^Bi: UTEVA + 50W × 8/pre-filter	Gamma spectrometry * LSC *	Chemical yield: 87 ± 3% for ^213^Bi, 95 ± 1% for ^68^Ga, 88 ± 2% for ^99m^Tc and 93 ± 3% for ^188^Re Turnover time: 19–58 min.	[34]
	^ 213 ^ Bi **	Parent radionuclide ^225^Ac	SI	Single sample	Anion exchange chromatography	-	Chemical yield: 85–93% Turnover time: 6 min.	[33]
Nuclear emergency preparedness	^ 89 ^ Sr, ^90^Sr	Milk	FI	Multi-sample (4 samples)	Cation exchange chromatography (Dowex 50W × 8 − 100) * Extraction chromatography (5 mL Sr resin)	LSC *	Chemical yield: 80% MDA: 0.7 Bq/L of ^89^Sr, 0.3 Bq/L of ^90^Sr Precision: 5% RSD Turnover time: <1 day	[26]
	^ 237 ^ Np, ^239^Pu	Urine (0.2–1 L)	LOV-SI	Single sample Renewable column	Extraction chromatography (ca. 300 mg TEVA resin, 100–150 µm)	ICP-MS *	Chemical yield: 88.7 ± 11.6% for Pu, 94.2 ± 2.0% for Np LOD: 1.0–1.5 pg/L for both ^237^Np and ^239^Pu Turnover time: 6 h	[65]
	^ 239 ^ Pu	Urine (1 L)	LOV-SI	Single sample Renewable column	Extraction chromatography (ca. 300 mg TEVA resin, 100–150 µm)	ICP-MS *	Chemical yield: > 90% LOD: 1.0–1.5 pg/L of ^239^Pu Turnover time: 6 h	[27]
Nuclear safeguards	^ 239 ^ Pu, ^240^Pu, ^241^Pu, ^242^Pu, ^244^Pu isotope ratios	Spiked working solution	FI	Single sample	Electrochemically modulated separation	ICP-MS	LOD: 0.055 fg of ^239^Pu Precision: 31.1% RSD for ^239^Pu/^244^Pu, 14.5% RSD for ^240^Pu/^244^Pu, 83.8% RSD for ^241^Pu/^244^Pu, 11.2% RSD for ^242^Pu/^244^Pu	[66]
Nuclear waste management	^ 90 ^ Sr	Aged nuclear waste samples from the Hanford site	SI	Single sample	Extraction chromatography (0.35 mL Sr resin)	Flow-through LSC	Chemical yield: 94 ± 5%. LOD: 2.62 Bq of ^90^Sr Turnover time: <40 min.	[67]
	^90^Sr, ^241^Am, ^99^Tc	Aged nuclear wastes	SI	Single sample Renewable column	Extraction chromatography (50 µL Sr resin, TRU resin and TEVA resin)	Flow-through LSC	Chemical yield: 92 ± 2% for ^90^Sr, 99 ± 5% for ^99^Tc	[68]
	^ 99 ^ Tc	Nuclear waste samples from the Hanford site	SI	Single sample	Extraction chromatography (0.83 mL TEVA, 20–50 µm)	Flow-through LSC	LOD: 2 ng of ^99^Tc Turnover time: 20–40 min.	[69]
	^ 99 ^ Tc	Nuclear waste simulant solutions and aged nuclear waste	SI coupling online microwave-assisted sample treatment	Single sample	Anion exchange chromatography (0.83 mL AG MP-1M, 38–75 µm)	Flow-through solid scintillator detector	-	[70]
	^ 99 ^ Tc	Nuclear waste simulant solutions and Hanford tank waste sample	SI coupling online microwave-assisted sample treatment	Single sample	Anion exchange column (AG MP-1M)	Flow-through solid scintillator detector	LOD: 23.5 kBq/L of ^99^Tc Precision: <10% RSD Turnover time: 12.5 min	[71]
	^99^Tc	Aged nuclear wastes	SI	Single sample Renewable column	Extraction chromatography (212 µL TEVA resin)	Flow-through LSC	LOD: 6 Bq/L Turnover time: 30 min	[72]
	^ 99 ^ Tc, ^230^Th, ^234^Th	Soil (0.25–5 g)	FI	Single sample	Extraction chromatography (ca. 30 mg TEVA resin and ca. 30 mg TRU resin)	ICP-MS	LOD: 11 Bq/kg of ^99^Tc, 3.7 Bq/kg of ^230^Th, 0.74 Bq/kg ^234^Th	[73]
	^ 230 ^ Th, ^233^U, ^239^Pu, ^241^Am	Spiked sample solution in 2 M HNO_3_	FI	Single sample	Extraction chromatography (0.63 mL TRU resin, 20–50 µm)	Flow- through LSC LSC * Alpha spectrometry *	Chemical yield: up to 102 ± 4% for ^241^Am up to 101 ± 3% for ^239^Pu up to 93 ± 4% for ^233^U up to 88 ± 3% for ^230^Th	[74]
	^ 237 ^ Np, ^242^Pu	Ground water at Gorleben site	FI	Single sample	Capillary electrophoresis	ICP-MS	LOD: 50 µg/L Turnover time: <15 min	[75]
	^ 237 ^ Np, ^238^Pu, ^239+240^Pu, ^241^Am	Dissolved vitrified nuclear waste	SI	Single sample	Extraction chromatography (0.63 mL TRU resin, 20–50 µm)	ICP-MS	U decontamination factor (for Pu determination): 3.0 × 10^5^	[76]
	^ 238 ^ Pu, ^239+240^Pu, ^241^Am, ^243+244^Cm, ^242^Cm	Vitrified glass waste, aged irradiated nuclear fuel and waste from Handford site	SI	Single sample	Extraction chromatography (0.63 mL TRU resin, 20–50 µm)	Flow-through LSC LSC * Alpha spectrometry *	Chemical yield: 85% for Pu, 86% for Am	[77]
Radioecology and tracer studies	^ 99 ^ Tc	Soil (1–10 g)	FI	Single sample	Tandem chromatography (0.75 mL TEVA resin and 0.17 mL TEVA resin)	ICP-MS	Chemical yield: 63–73% LOD: 50 mBq/L Precision: <4% RSD Turnover time: 3–5 h	[78]
	^ 236 ^ U	Seawater (10 L)	FI	Multi-sample (4 samples)	Extraction chromatography (2 mL UTEVA resin, 100–150 µm)	ICP-MS * AMS *	Chemical yield: 80−100% LOD: 6.6 × 10^−11^ of ^236^U/^238^U atomic ratio Turnover time: 4 h	[79]

* Offline separation or measurement. ** The flow system is used for the radionuclide purification. Abbreviations: AMS: accelerator mass spectrometry; DF: decontamination factor; FI: flow injection; ICP-MS: inductively coupled plasma mass spectrometry; HPIC: high performance liquid chromatography; LBPC: low background proportional counter; LIS: lab-in-syringe; LLD: lower limit of detection; LLME: liquid–liquid microextraction; LOD: limit of detection; LOV: lab-on-valve; LSC: liquid scintillation counting; LWCC: long waveguide capillary cell; MSFIA: multi-syringe flow injection analysis; MSA: magnetic-stirring-assisted; MDA: minimum detectable activity; SI: sequential injection.
